# Effects of Classroom Ventilation Rate and Temperature on Students’ Test Scores

**DOI:** 10.1371/journal.pone.0136165

**Published:** 2015-08-28

**Authors:** Ulla Haverinen-Shaughnessy, Richard J. Shaughnessy

**Affiliations:** 1 Indoor Air Program, the University of Tulsa, 600 S College Ave, Tulsa, OK, 74104, United States of America; 2 National Institute for Health and Welfare, Department of Health Protection, P.O. Box 95, 70701, Kuopio, Finland; Columbia University, UNITED STATES

## Abstract

Using a multilevel approach, we estimated the effects of classroom ventilation rate and temperature on academic achievement. The analysis is based on measurement data from a 70 elementary school district (140 fifth grade classrooms) from Southwestern United States, and student level data (N = 3109) on socioeconomic variables and standardized test scores. There was a statistically significant association between ventilation rates and mathematics scores, and it was stronger when the six classrooms with high ventilation rates that were indicated as outliers were filtered (> 7.1 l/s per person). The association remained significant when prior year test scores were included in the model, resulting in less unexplained variability. Students’ mean mathematics scores (average 2286 points) were increased by up to eleven points (0.5%) per each liter per second per person increase in ventilation rate within the range of 0.9–7.1 l/s per person (estimated effect size 74 points). There was an additional increase of 12–13 points per each 1°C decrease in temperature within the observed range of 20–25°C (estimated effect size 67 points). Effects of similar magnitude but higher variability were observed for reading and science scores. In conclusion, maintaining adequate ventilation and thermal comfort in classrooms could significantly improve academic achievement of students.

## Significance

We studied relationships between students’ test scores and both classroom ventilation rate and temperature. The study is unique, because it utilizes multilevel analyses and a large database, including measured data on ventilation and thermal parameters, and student level data on standardized test scores. Based on the results, maintaining adequate ventilation and thermal comfort could raise an average tests score to “commended performance”. The study helps to understand the potential benefits of effectively managing indoor environmental factors in schools.

## Introduction

Recent studies have reported associations between provision of ventilation (outdoor air) and students’ health and academic performance. For example, one field study from California found a statistically significant 1.6% reduction in illness absence per each additional liter per second per person (l/s per person) of ventilation provided [1]. Another study from the Southwestern United States estimated that for every l/s per person increase up to 7.1 l/s per person, the percentage of students passing the State’s core curriculum based standardized tests could increase by 2.9% (95%CI 0.9–4.8%) in mathematics and 2.7% (0.5–4.9%) in reading [2]. At the same time, these studies reported average ventilation rates of the school systems studied being equal or less than 4 l/s per person, indicating that the majority of schools had ventilation rates below the American Society of Heating, Refrigerating, and Air-Conditioning recommended minimum of 7.1 l/s per person [3]. Also experimental data from Denmark associated increased ventilation rates in classrooms with improved school performance [4]. Low ventilation rates can result in an increased exposure to indoor air pollutants, assumed to be the primary reason for adverse effects on occupant health and performance [5–7].

In addition to inadequate ventilation, some studies have associated elevated indoor temperatures in schools with impaired performance [4, 8]. ASHRAE [9] recommends indoor temperatures in the winter be maintained between 20 and 24°C (68–75°F), whereas summer temperatures should be maintained between 23 and 26°C (73–79°F). These ranges are prescribed acceptable for sedentary or slightly active persons. Both measured ventilation rates and elevated temperatures have been associated with students’ self-reported stuffiness or poor indoor air quality in classrooms [10].

The majority of previous studies have been case studies [4] or cross-sectional studies based on school or grade level data on students’ background, absenteeism, and performance [2, 11]. At present, we are not aware of previous school effect studies analyzing student level data on performance as well as measured data on ventilation and thermal parameters with multilevel models, which take into consideration the nested structure of the data, i.e. the basic assumption that pupils attending the same school (and classroom) are in some respects more alike than pupils from two different schools (and classrooms). Also the fact that the pupils come from different socioeconomic backgrounds could explain variations in their health and school achievement, which should be taken into account when assessing the amount of variation in pupil outcomes conditioned by differences between schools [12]. Herewith, we report findings from multilevel analyses using linear mixed models (LMM), aiming to study the effects of ventilation and temperature on test scores. The underlying null hypothesis is that students’ test scores are not affected by their classroom ventilation rate or temperature.

## Materials and Methods

Seventy elementary schools in Southwestern US School district were surveyed and monitored for multiple IEQ parameters during the academic year of 2008–2009. Prior to the data collection, the research project applied and obtained an approval from the school district, with the condition of maintaining the confidentiality of the school district’s participation in the study. The area climate is characterized by hot summers (an average of 107 days with maximum temperature ≥ 32°C; 90°F) and mild winters (an average of 19 days with minimum temperature below freezing). The average annual temperature is about 20°C (69°F). During the school year, the 5-year average heating degree days is about 946 in°C HDD (1783°F HDD) for base temperature of 18°C (65°F) and cooling degree days is about 530°C CDD (1000°F CDD) for base temperature of 23°C (73°F).

Monitoring of indoor temperature (T), relative humidity (RH), and carbon dioxide (CO_2_) was conducted by fourteen TSI QTrak Monitors. The monitors used were calibrated according to the instruction manual and intercalibrated (i.e., compared with each other) weekly. The monitors were rotated on a weekly basis to seven new schools between January 26 and April 18, 2009. In each school, the monitors were deployed in two separate 5th grade classrooms on a Monday morning and picked up on Friday afternoons. Therefore, the continuous data logging lasted a minimum of four days in each classroom. The data loggers recorded data in 5-min increments throughout the days.

Classrooms were monitored under closed conditions, i.e. keeping windows and doors closed as best possible during the occupied hours. Heating, ventilation, and air conditioning (HVAC) systems were operated with fans in the on position during the monitoring period. Recognizing that seasonal times of the year will have some impact on ventilation rates, the closed classroom conditions were instilled to provide an estimate of ventilation rates based on mechanical system introduction of outdoor air.

Preliminary analyses included assessment of indoor T and RH data over a school day, matched with hourly outdoor data obtained from the closest weather station. Average, minimum, and maximum values during the occupied school hours were estimated for each classroom [13]. The following analyses were focused on ventilation rate and average indoor T during the occupied school hours in the classrooms. Ventilation rates for each classroom monitored were estimated from CO_2_ data as described by Haverinen-Shaughnessy et al. [2], using a peak-analysis approach based on a mass-balance model [14, 15]. Briefly, since the studied classrooms were 5th grade classrooms and had similar occupant density and activity conditions, the CO_2_ generation rate used was 0.0043 l/s per person for students, whereas 0.0052 l/s per person rate was used for teachers [16–18]. The peak-analysis approach assumes that CO_2_ concentrations reaches steady state (C_eq_) in the classrooms. The peak concentration of CO_2_ recorded during the measurement period was used as the steady-state value of CO_2_.

Other classroom level data included highest degree held by the teacher, which were obtained from the district. In addition, student individual data for 2008–2009 school year were obtained to profile each fifth grade student (N = 3109) in the 70 schools (140 classrooms) related to the student’s gender, ethnic background, participation in the free lunch program (commonly used as a socioeconomic indicator), English language proficiency, “gifted” status (i.e. a student who has demonstrated potential abilities of high performance), and mobility rate, as well as data related absenteeism and absenteeism due to illness (corresponding to number of days absent). The information on the students was blinded to the researchers, as it was coded and anonymized.

The district also provided data from the statewide assessment of learning. These data includes students’ individual (coded and anonymized) test scores in mathematics, reading, and science from assessment performed in the spring of 2009. In addition, test scores from previous school year (spring 2008) were obtained for mathematics and reading. The annual assessment is designed to relate levels of test performance to the expectations defined in the state-mandated curriculum standards. The state used scale score of 2100 for ‘met standard’ and 2400 for ‘commended performance’ for all subject areas.

IBM SPSS statistical package version 21 was used for data analyses. Using linear mixed modelling, the school and classroom or teacher intercept terms were used to account for the dependence among the children at the same school. The model with the random effects (school and classroom or teacher) was used as the zero-model. Final model included random effects and both student and school level variables fitted to the model one by one. The continuous ventilation rate and indoor T variables were centered around their grand means. Since absenteeism and absenteeism due to illness were significantly correlated, a composite variable ‘number of days absent, no illness’ (i.e. ‘total days absent’ minus ‘number of days absent due to illness’) was formed, and the Akaike information criterion (AIC) was used to determine which variables were most suitable for the model. After fitting each variable, the model was studied for within and between subject variance components (as compared to zero-model) and intraclass correlation (ICC), which represents the proportion of total variance that occurs between schools, while the remaining proportion represents variance among students within schools [19]. We also computed the effects of ventilation rate and indoor T on the variance component between schools, to estimate which proportion of the explainable variation in the school mean test scores could be explained by these two factors.

R version 3.1.0 (lme4, LMERConvenienceFunctions, and effects packages) was used to estimate the overall effect size (range) of ventilation rate and indoor T and to illustrate the partial effect of ventilation rate on mathematics score for indoor T below and above the observed mean value. Functions PlotLMER.fnc and effect produce and plot partial effects of a linear-mixed effects-model fitted with lmer (compatible with package lme4).

## Results and Discussion

All of the studied classrooms were equipped with locally controlled mechanical HVAC systems. The ventilation systems in 44 schools consisted of single-zone individual room units (i.e., residential style up flow furnace-type systems and side-wall mounted unit ventilators). In addition, 15 schools had fan coil units which were mounted in the individual classrooms for heating and cooling purposes, but no outdoor air provision, and 12 schools had multi-zone air handling units (primarily consisting of rooftop units and central packaged units that would serve two to four classrooms in the building). The multi-zone units would serve classrooms with similar occupant density and occupant activity conditions. While windows could be opened in the majority of the classrooms studied, it was reported that 76% of the classrooms did not open the widows on a daily basis, which was compatible to the district’s overriding policy to maintain classrooms with windows closed in order to rely on the mechanical system to temper and condition the air, and to maintain a controlled environment.

The mean classroom level ventilation rate was 3.6, 95%CI 3.2–4.0 l/s per person, and the mean indoor T was 23°C, 95%CI 22.6–22.9°C (73°F, 95%CI 72.6–73.3°F). The mean school level mathematics score was 2286 (95%CI 2258–2313) and the proportion of total variance (ICC) occurring between subjects (school * classroom) was 0.21 (21%). Final multivariate model for mathematics included the following student level variables: gifted status, limited English proficiency, ethnic group, mobility, eligibility to free or reduced lunch, gender, the composite variable ‘number of days absent, no illness’, as well as teacher’s highest degree, and ICC related to this model (not including ventilation rate or indoor T) was 0.10.

After including classroom ventilation rate and indoor T in the final model, ICC decreased to 0.09. Therefore, about 10% of the defined variation in mean mathematics scores between subjects could be explained by ventilation rate and indoor T. Based on the final model, subjects exposed to a difference of 1 l/s per person in ventilation rate differed by 7 (95%CI 1–12) points in mathematics scores; whereas subjects exposed to a difference of 1°C (1.8°F) in indoor T differed by 13 (95%CI 1–26) points, correspondingly (Table 1). The interaction ventilation rate * indoor T was not statistically significant (parameter estimate -3, 95%CI -8-3), possibly due to limited sample size.

**Table 1:** Descriptive statistics, parameter estimates for each fixed effect individually, and final model estimates for mathematics score^a^.

			Estimates for each predictor^b^ individually			Final model^c^		
	N	%	Estimate	(95% CI)	Sig	Estimate	(95% CI)	Sig
Predictors								
Intercept						**3064.9**	**2555.0–3574.7**	**.000**
Gifted status								
No	2811	90.4	-265.8	-(292.6–239.1)	.000	**-228.0**	**-(253.9–202.0)**	**.000**
Yes	298	9.5	0^d^	.	.	**0** ^d^	.	.
Limited English Proficiency								
No	2408	77.5	134.9	110.4–159.4	.000	**126.3**	**103.4–149.2**	**.000**
Yes	684	22.0	0^d^	.	.	**0** ^d^	.	.
Ethnic group								
Native American	5	0.2	-30.5	-228.3–167.3	.763	**16.0**	**-185.7–217.7**	**.876**
Asian	132	4.2	67.0	21.9–112.2	.004	**66.9**	**23.9–110.0**	**.002**
African American	394	12.7	-201.9	-(233.8–170.0)	.000	**-155.0**	**-(185.4–124.5)**	**.000**
Hispanic	1824	58.7	-129.7	-(153.7–105.7)	.000	**-68.0**	**-(92.0–44.0)**	**.000**
Caucasian	754	24.3	0^d^	.	.	**0** ^d^	.	.
Mobility								
Moved to a different district between the fall and spring	232	7.5	-131.7	-(165.7–97.7)	.000	**-98.1**	**-(128.9–67.3)**	**.000**
Moved to a different school between the fall and spring	118	3.8	-90.7	-(134.8–46.6)	.000	**-60.6**	**-(100.6–20.7)**	**.003**
Stayed the whole year	2758	88.7	0^d^	.	.	**0** ^d^	.	.
Eligibility to free or reduced lunch								
Free lunch	1696	54.6	-140.5	-(164.2–116.9)	.000	**-52.9**	**-(76.0–29.7)**	**.000**
Reduced lunch	220	7.1	-89.2	-(125.2–53.2)	.000	**-37.0**	**-(70.5–3.6)**	**.030**
Not eligible	1193	38.4	0^d^	.	.	**0** ^d^	.	.
Gender								
Male	1597	51.4	-10.2	-26.7–6.2	.223	**1.8**	**-13.1–16.6**	**.813**
Female	1512	48.6	0^d^	.	.	**0** ^d^	.	.
Teacher’s highest degree								
Bachelor’s degree	105	74.5	-19.3	-67.6–29.0	.431	**-31.9**	**-(62.5–1.4)**	**.041**
Master’s degree	36	25.5	0^d^	.	.	**0** ^d^	.	.
		Mean						
Total days absent	3108	5.9	-4.0	-(5.3–2.6)	.000	**-**		
Days absent due illness	3108	2.2	-0.5	-2.9–2.0	.722	**-**		
Days absent no illness	3108	3.8	-8.1	-(10.1–6.1)	.000	**-6.8**	**-(8.6–4.9)**	**.000**
Ventilation rate[l/s per person]	3092	3.6	19.7	11.4–28.0	.000	**6.7**	**1.0–12.4**	**.022**
Indoor T [°C]	3040	22.7	-32.8	-(51.6–13.9)	.001	**-13.4**	**-(25.9–0.9)**	**.036**

^**a**^ Dependent Variable

^b^ added to the zero-model

^c^ Includes all predictors

^d^ This parameter is set to zero because it is redundant

Inclusion of previous year’s test score did not result in removal of the other variables selected, and it did not change the parameter estimates for ventilation rate (Table 2). This model was better in predicting the mathematics score: the variance component within subjects diminished by 43% and the variance component between subjects diminished by 30%. Many student characteristics and test-taking ability, which could affect each student’s test scores, should be accounted for by inclusion of previous year’s test scores; however, there remains residual confounding, which could be related to not being able to account for information on unmeasured variables. Such information includes any changes in each individual student’s conditions since the previous year’s test that could influence his/her performance.

**Table 2:** Estimates for ventilation rate and indoor T based on two alternative models^a^.

Description of the model		Estimates for each predictor^b^ individually			Final model		
Alternative models	Mean	Estimate	95% CI	Sig	Estimate	95% CI	Sig
**Mathematics score 2007–2008 included** ^**c**^							
Mathematics score 2007–2008, N = 2675^d^	2263.5	.9	.8-.0	.000			
Ventilation rate [l/s per person], N = 2661	3.7	19.5	11.0–28.1	.000	**6.7**	**2.0–11.3**	**.006**
Indoor T [°C], N = 2611	22.7	-33.2	-(52.5–13.9)		**-4.2**	**-14.5–6.1**	**.420**
**Ventilation rates > 7.1 l/s per person filtered** ^**e**^							
Ventilation rate [l/s per person], N = 2951	3.3	36.3	23.7–48.8	.000	**11.2**	**2.0–20.4**	**.017**
Indoor T [°C], N = 2899	22.8	-33.6	-(52.6–14.7)	.001	**-12.0**	**-24.7–0.6**	**.062**

^a^ Dependent Variable: Mathematics score

^b^ added to the zero-model

^c^ Includes previous year’s mathematics score added to the models shown in Table 1.

^d^ Note: previous year’s mathematics score is not available for all students, which could be related to mobility

^e^ Final model as in Table 1 (data related to classrooms with ventilation rates > 7.1 l/s per person filtered)

**Fig 1:**
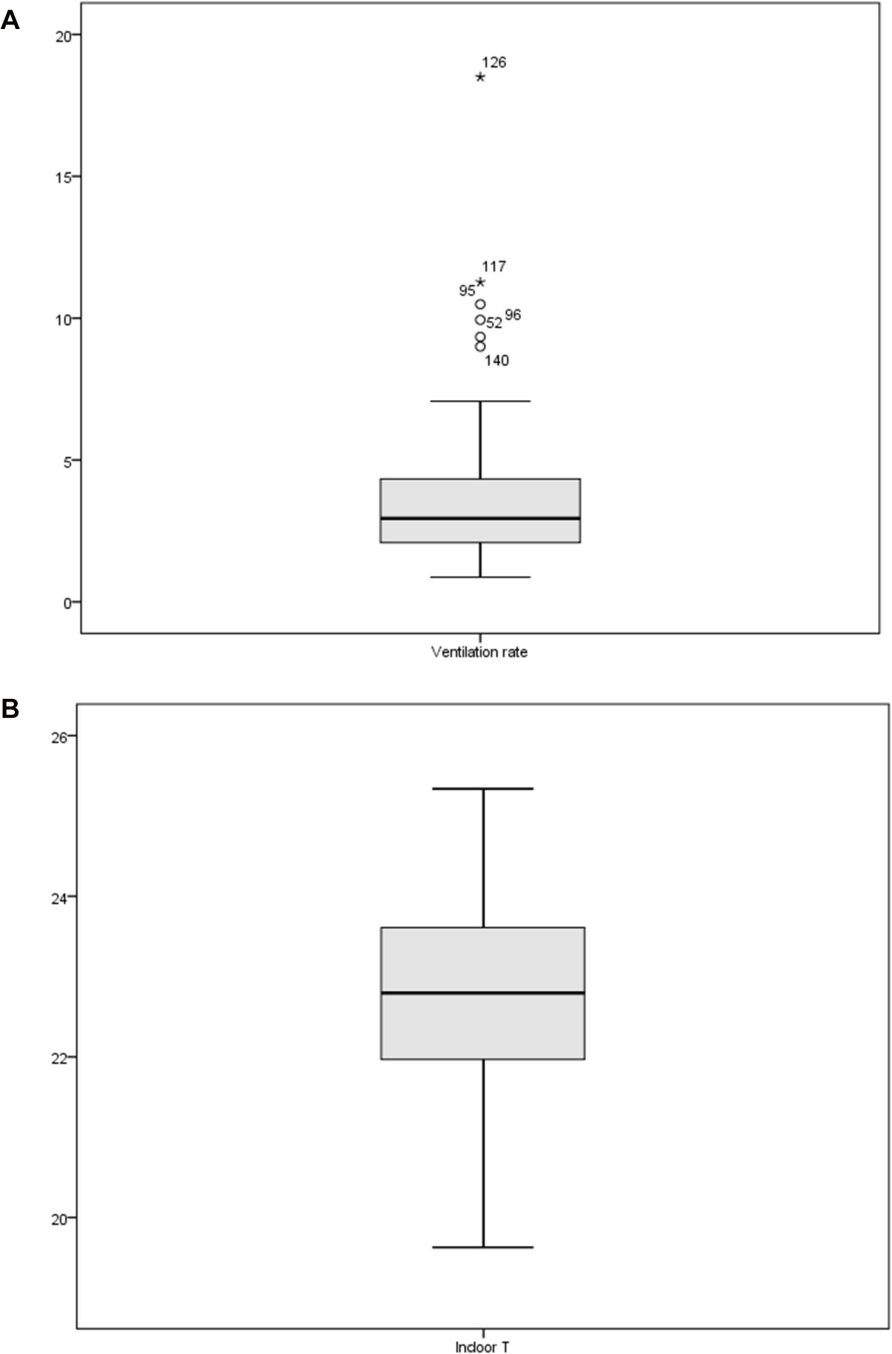
Box plots for ventilation rate [l/s per person] and indoor T [°C]. The outliers are labeled with case numbers.

Ventilation rates >7.1 l/s per person (i.e. meeting the recommended minimum) exceeded 1.5 times the interquartile range, hence indicated as outliers in the boxplot (Fig 1).

**Fig 2:**
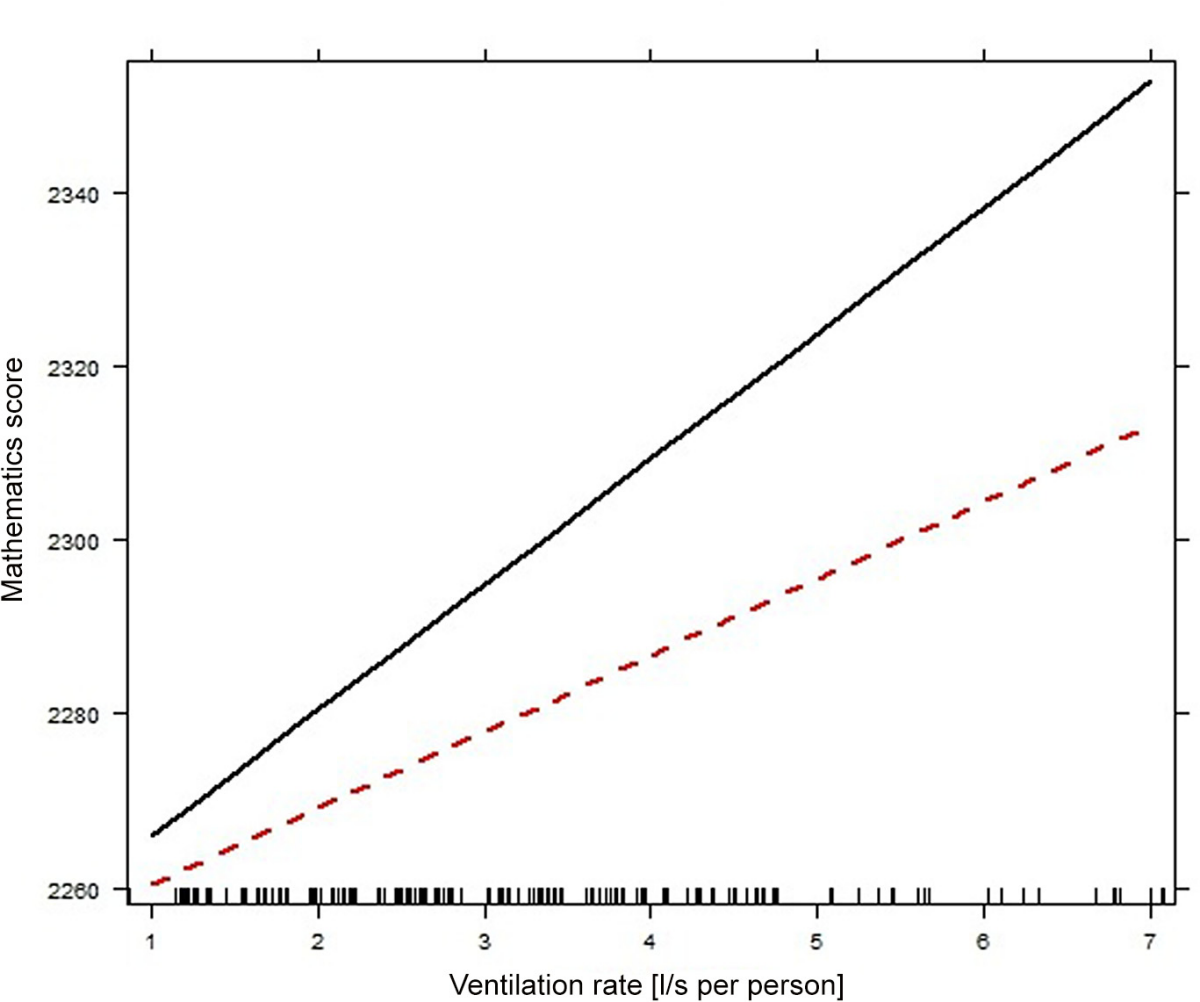
Partial effect of ventilation rate on mathematics score for indoor T below and above 23°C (73°F). Solid line corresponds with indoor T below 23°C (73°F).

As shown in Table 2, filtering these six classrooms (housing 140 students from five schools), resulted in the parameter estimate for ventilation rate increasing to 11 (95%CI 2–20), whereas the parameter estimate for indoor T did not change considerably. The absolute value of parameter estimate for interaction increased, but remained non-significant (parameter estimate -4, 95%CI -13-4). The result concurs with a previous study that found a stronger, statistically significant linear association between ventilation rate up to 7.1 l/s per person and the proportion of students passing standardized tests in mathematics [2]. Thus, it appears that the small number of classrooms meeting the recommended minimum ventilation rate increases the uncertainty in the results when schools with higher ventilation rates are included. Corresponding to this model, the estimated effect size (range) for ventilation rate (up to 7.1 l/s per person) was 74 points, and for indoor T it was 64 points. Therefore, these effects combined (138 points) could raise an average tests score (2286 points) to ‘commended performance’ (>2400 points). Fig 2 illustrates the partial effects of ventilation rate for indoor T below and above the observed mean value.

Further on, we performed stratified analyses for based on the gifted status, English language proficiency, as well as the largest free lunch eligibility categories and ethnic groups (Table 3). With respect to ventilation rate, the final model estimates for the association between mathematics scores were within the whole population standard error (5 points) across different groups, except among the group of African American students, where the estimate was 9 points higher. With respect to indoor T, the estimates were also within the whole population standard error (about 6 points), except among the students with limited English proficiency, where the estimate was 18 points higher, and among the groups of gifted students and African American students, where the associations were positive (higher temperature corresponded with higher test scores) but not statistically significant.

**Table 3:** Final model^a^ estimates for mathematics score stratified for three largest ethnic groups^b^ (Hispanic, Caucasian and African American), eligibility to free lunch^c^, gifted status^d^, and limited English proficiency^e^.

Sample	Whole	Ethnic group			Eligibility to free lunch		Gifted		English	
	population	Hispanic	Caucasian	African American	Free lunch	Not eligible	No	Yes	Limited	Proficient
N	2951	1717	718	382	1633	1109	2811	298	684	2408
	Estimate	Estimate	Estimate	Estimate	Estimate	Estimate	Estimate	Estimate	Estimate	Estimate
	95%CI	95%CI	95%CI	95%CI	95%CI	95%CI	95%CI	95%CI	95%CI	95%CI
Ventilation rate	11.2*	9.6	11.3	20.4*	6.7	13.8**	11.2*	15.7*	6.4	11.2*
[l/s per person]	2.0–20.4	-2.2–21.4	-0–22.6	2.1–38.7	-6.3–19.7	4.9–22.8	1.6–20.8	2.9–28.5	-12.9–25.8	1.5–20.9
Indoor T [°C]	-12.0	-18.4*	-13.8	7.3	-10.3	-12.7	-12.8	2.9	-30.3*	-7.1
	-24.7–0.6	-(34.1–2.6)	-30.5–2.9	-18.2–32.8	-27.0–6.4	-26.2-.8	-26.0-.5	-15.7–21.5	-(55.7–5.0)	-20.6–6.3

* p < 0.05

**p< 0.01

*** p<0.001

^a^ Ventilation rates > 7.1 l/s per person filtered

^b^ Final model includes gifted status, limited English proficiency, mobility, eligibility to free or reduced lunch, gender, teachers’ highest degree, and days absent no illness

^c^ Final model includes gifted status, limited English proficiency, ethnic group, mobility, gender, teachers’ highest degree, and days absent no illness

^d^ Final model includes limited English proficiency, ethnic group, mobility, eligibility to free or reduced lunch, gender, teachers’ highest degree, and days absent no illness

^e^ Final model includes gifted status, ethnic group, mobility, eligibility to free or reduced lunch, gender, teachers’ highest degree, and days absent no illness

Previous studies have observed differences between Caucasian, Hispanic, and African American students in terms of temperature preference while learning [20, 21], which could indicate possibility for effect modification by ethnic background. However, the sample size in the current study appears limited to further explore this possibility. Further studies are also needed to determine if classroom temperature has a larger effect on students with limited language proficiency.

**Fig 3:**
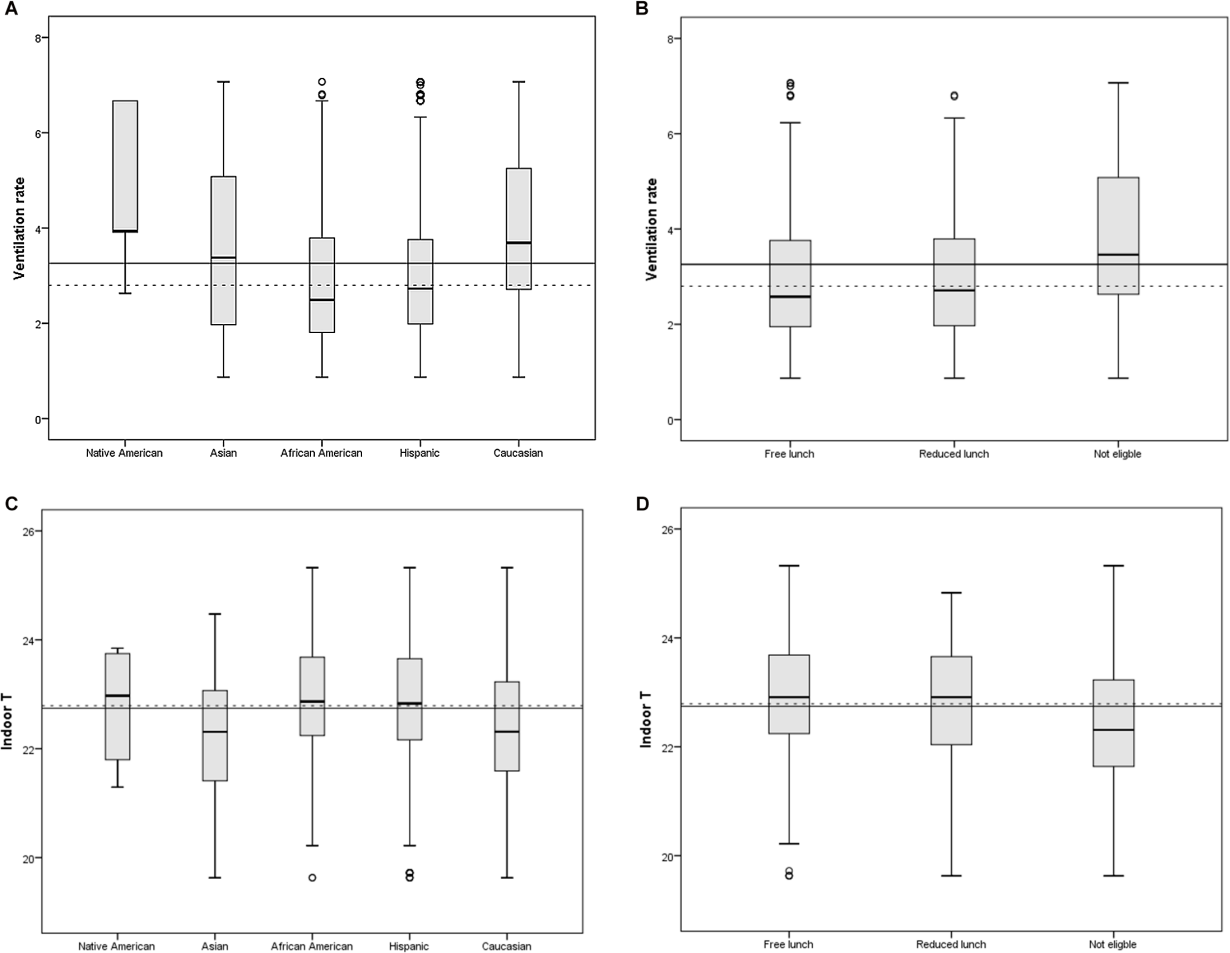
Box plots for ventilation rate [l/s per person] and indoor T [°C] by ethnic group and eligibility for free lunch. Solid line corresponds with population means and dotted line with medians.

We also checked if class size (i.e. number of students in the classroom) and school level socioeconomic variables (e.g. percent of student eligible for free lunch) should be included in the models in addition to student-level variables as they have been shown to be important predictors of achievement in previous studies [12, 22]. While these variables did not meet the model selection criteria, we observed significant correlations between school level socioeconomic variables and both ventilation rate and indoor T (data not shown). In addition to making it more difficult to separate the effects of different variables, such correlations may point toward possible inequity issues. Fig 3 shows box plots for ventilation and indoor T by ethnic group and eligibility for free lunch, indicating that on the average, African American and Hispanic students, as well as free lunch eligible were exposed to lower ventilation rates and higher temperatures.

As shown in Table 4, correlations between mathematics, reading, and science scores were high.

**Table 4:** Pearson correlations between test scores.

		Mathematics 2008–2009	Mathematics 2007–2008	Reading 2008–2009	Reading 2007–2008	Science 2008–2009
Mathematics 2008–2009	Pearson Correlation	1	.74**	.64**	.63**	.70**
	N	3017	2653	2977	2645	2984
Mathematics 2007–2008	Pearson Correlation	.74**	1	.62**	.69**	.65**
	N	2653	2675	2629	2642	2636
Reading 2008–2009	Pearson Correlation	.64**	.61**	1	.75**	.68**
	N	2977	2629	2988	2622	2952
Reading 2007–2008	Pearson Correlation	.63**	.69**	.75**	1	.68**
	N	2645	2642	2622	2669	2631
Science 2008–2009	Pearson Correlation	.70**	.65**	.68**	.68**	1
	N	2984	2636	2952	2631	3004

**. Correlation is significant at the 0.01 level (2-tailed).

The associations between ventilation rate and indoor T and reading and science scores were similar to those related to mathematics scores (Table 5). Yet, adding student level variables to the zero-model for reading reduced ICC from 0.28 to 0.08, after which adding ventilation rate and indoor T did not change it. Adding previous years reading score resulted in ICC decreasing to 0.04, while variance component within subjects diminished by 36% and the variance component between subjects diminished by 69%. Adding student level variables to the zero-model for science reduced ICC from 0.26 to 0.07 and adding ventilation rate and indoor T reduced it further by 8%. There were statistically significant associations between gender and both reading and science: girls achieved higher scores in reading, whereas boys achieved higher scores in science. Teacher’s degree did not appear to affect reading scores whereas it associated with both mathematics and science scores.

**Table 5:** Final model estimates for fixed effects, reading and science.

	Reading score ^a^			Science score ^a^		
Model	Final			Final		
Parameter	Estimate	95% CI	Sig	Estimate	95% CI	Sig
Intercept	2762.7	2333.4–3192.0	.000	3011.7	2555.1–3468.2	.000
Gifted status						
No	-173.3	-(195.6–151.0)	.000	-232.3	-(258.0–206.5)	.000
Yes	0^b^	.	.	0^b^	.	.
Limited English Proficiency						
No	114.3	94.7–134.0	.000	151.9	129.6–174.2	.000
Yes	0^b^	.	.	0^b^	.	.
Ethnic group						
Native American	11.4	-160.9–183.8	.897	-120.1	-319.7–79.6	.239
Asian	-32.4	-69.2–4.3	.084	-11.8	-54.2–30.6	.585
African American	-133.0	-(159.2–106.8)	.000	-187.9	-(217.8–157.9)	.000
Hispanic	-85.9	-(106.5–65.3)	.000	-108.5	-(132.2–84.8)	.000
Caucasian	0^b^	.	.	0^b^	.	.
Mobility						
Moved to a different district between the fall and spring	-56.0	-(83.4–28.5)	.000	-59.2	-(89.2–29.3)	.000
Moved to a different school between the fall and spring	-45.0	-(79.3–10.8)	.010	-72.8	-(111.7–34.0)	.000
Stayed the whole year	0^b^	.	.	0^b^	.	.
Eligibility to free or reduced lunch						
Free lunch	-76.4	-(96.2–56.6)	.000	-80.0	-(102.6–57.4)	.000
Reduced lunch	-70.0	-(98.7–41.3)	.000	-67.5	-(100.3–34.6)	.000
Not eligible	0^b^	.	.	0^b^	.	.
Gender						
Male	-27.6	-(40.4–14.9)	.000	44.4	29.6–59.1	.000
Female	0^b^	.	.	0^b^	.	.
Teachers’ highest degree						
Bachelor’s degree	-9.8	-35.5–15.9	.452	-26.6	-53.9-.8	.057
Master’s degree	0^b^	.	.	0^b^	.	.
Days absent no illness	-3.1	-(4.7–1.6)	.000	-4.6	-(6.5–2.8)	.000
Ventilation rate [l/s per person]	4.3	-.5–9.1	.078	4.6	-.5–9.7	.074
Indoor T [°C]	-7.4	-18.0–3.1	.167	-12.4	-(23.5–1.2)	.031
Ventilation rates ≥ 7.1 l/s per person filtered						
Ventilation rate [l/s per person]	16.0	8.9–23.1	.000	11.1	3.0–19.1	.008
Indoor T [°C]	-6.5	-16.3–3.2	.189	-11.3	-(22.5-.2)	.046

^a^ Dependent variables

^b^ This parameter is set to zero because it is redundant

Overall, ICC between subjects was higher for reading and science than for mathematics, but with respect to reading, inclusion of the student level variables diminished the variance components more effectively; whereas the school level variables (ventilation rate, indoor T, and teacher’s degree) appeared to be more relevant for mathematics and science. The associations between the school level variables and test scores were of similar magnitude. However, it should be noted that many other studies have not found evidence that a master’s degree improves teacher skills, attributing the main effects of teacher quality to other characteristics, data not available for this study [22].

In this study, illness absence was not associated with the tests scores. On the contrary, separating non-illness based absence from total days absent resulted in stronger associations, leading to selection of non-illness based absence to the final models. A possible explanation is that motivated students can catch up with their school work after recovering from short-term illnesses. However, other types of absence, which are unlikely related to indoor environmental quality in classrooms, may be more difficult to overcome. These types of absences have been linked to students who will not attend school to avoid bullying, unsafe conditions, harassment and embarrassment, and students who do not attend school because they (or their parents), do not see the value in being there [23]. We also checked if ventilation rates could be associated with illness absence as suggested by Mendell et al. [1], however, we could not confirm this finding. It appears that in these data, the relationship between ventilation rates and test scores is caused by other mechanism(s), such as decreased decision making performance [24], or neurologic symptoms, such as headache, confusion, difficulty thinking, difficulty concentrating, or fatigue [7], and not by increasing illness absence.

It should be noted that this study was conducted all in one grade level and one school district, state, and climate. These restrictions are useful for controlling variability and for increasing precision, but caution is necessary in extrapolating to other types of age groups, school systems, and climates. In addition, the estimates for ventilation rates and temperatures were drawn from a relatively short measurement period. Continuing the study, we collected data over the following school year in a sub-sample of 27 schools, and observed a high correlation (0.791, p<0.001) between ventilation rates estimated based on data in the springs of 2009 and 2010, indicating that the measured ventilation rates in majority of schools could be representative of a long term situation [13].

There exists some uncertainty in the ventilation rate estimates since in many classrooms, the steady state concentrations were not actually attained due to the ventilation rates being so low. In these classrooms, the estimated ventilation rate derived from the peak-analysis approach may reflect an overestimation of the actual ventilation rate. Additional uncertainty is related to the calculation of a CO_2_ source generation, which was based on several factors such as age, assumed body weight and surface area, and level of physical activity (light activity). The activity typically varies throughout a school day: higher activity would mean that the actual ventilation rates were lower than the estimated values. On the other hand, windows were asked to be kept closed during the monitoring period, which could result in underestimating the ventilation rate in classrooms with operable windows. In effect, the estimated ventilation rates, while windows were closed, were those afforded by the mechanical system in place. However, it was reported that majority of classrooms were not opening windows on a daily basis. Also considering that the district’s policy does not encourage opening windows, the approach used for monitoring with windows closed in this study is most representative.

There were no statistically significant correlations between ventilation rate and average indoor or outdoor T. There was only a weak positive correlation between indoor and outdoor average temperatures (Spearman correlation .243, p<0.05), indicating that indoor T is relatively independent on the outdoor conditions, and could be more reflective of the individual school building and its heating and cooling system operation. Including outdoor T in the LMM models did not change the results considerably (data not shown).

With respect to the temperature findings, considering that there are different thermal comfort envelopes for different seasons, it appears that the schools fulfilled the recommendations. The observed associations would indicate that the higher temperatures (for example as specified for summer) might not be ideal for school buildings where students are expected to learn and perform.

Heating, ventilation, and air conditioning are responsible for a large part of school buildings’ operation costs as well as their carbon footprint. From both an economic and environmental points of view, schools should strive for optimal HVAC operation to keep energy consumption in check. In support of this premise, US EPA [25] has estimated that a traditional system, upgraded with inclusion of a modern energy recovery ventilation system, can allow for an increased ventilation rate from 2.4 l/s (or 5 cfm) per person to 7.1 l/s (or 15 cfm) per person, with no negative implications in terms of first cost, energy costs, and moisture control.

In conclusion, we could not reject an alternative hypothesis that students’ test scores may be affected by their classroom ventilation rate and temperature. Further studies (including interventions) are needed in order to examine the causality of the observed relationships, the residual confounding, and whether the results can be generalized to other climates, building types, and HVAC modes.
